# A Comparative Study of Clinical and Demographic Profiles of Multiple Sclerosis Patients in Two Regional Centers in Denmark and Romania

**DOI:** 10.3390/neurolint18020025

**Published:** 2026-01-29

**Authors:** Oana Vrînceanu, Rodica Bălașa, Smaranda Maier, Luigi Pontieri, Melinda Magyari

**Affiliations:** 1 Doctoral School, “George Emil Palade” University of Medicine, Pharmacy, Science and Technology of Targu Mures, 540142 Targu Mures, Romania; oana.mosora@umfst.ro; 2 Department of Neurology, “George Emil Palade” University of Medicine, Pharmacy, Science and Technology of Targu Mures, 540136 Targu Mures, Romania; smaranda.maier@umfst.ro; 3 Ist Neurology Clinical, Emergency Clinical County Hospital Targu Mures, 540136 Targu Mures, Romania; 4 The Danish Multiple Sclerosis Registry, Department of Neurology, Copenhagen University Hospital—Rigshospitalet, Glostrup, DK-2600 Copenhagen, Denmark; luigi.pontieri@regionh.dk (L.P.); melinda.magyari.01@regionh.dk (M.M.); 5 Danish Multiple Sclerosis Center, Department of Neurology, Copenhagen University Hospital—Rigshospitalet, Glostrup, DK-2600 Copenhagen, Denmark; 6 Institute for Clinical Medicine, University of Copenhagen, Blegdamsvej 3B, 33.5, Section A, DK-2200 Copenhagen N, Denmark

**Keywords:** multiple sclerosis, DMSC, DMTs, epidemiology

## Abstract

**Background**: Environmental factors are known to influence the clinical presentation of patients with multiple sclerosis. This study aims to compare the demographic and clinical characteristics of multiple sclerosis patients treated at two diverse geographical settings. **Methods**: A cross-sectional, observational cohort study was conducted in two MS centers: the Danish Multiple Sclerosis Center (DMSC) in Copenhagen, Denmark and the Regional MS Center in Târgu Mureș, Romania. We compared patients’ demographic and clinical characteristics between MS centers, including sex distribution, current age, MS onset age, latest EDSS scores, symptomatology at disease onset, MS phenotype and type of ongoing DMT. **Results**: In both cohorts, sex distribution was similar, with females constituting 69.2% in DMSC, and 65.7% in Târgu Mureș. Pyramidal symptoms at MS onset were predominant among Targu Mures patients (32.7%), while sensory symptoms were more frequent among DMSC patients (33%). Progressive forms of MS were more prevalent in Târgu Mureș (22.6%) compared to DMSC (9.9%). High-efficacy DMTs were on use by 58.3% patients in DMSC and only by 29.4% patients in Târgu Mureș, who were mostly on low-efficacy DMTs (54.4% vs. 12.4% in DMSC). **Conclusions**: The study highlights both shared and distinct characteristics of MS patients treated in these two centers. These findings underscore the importance of regional considerations in the management and treatment of MS.

## 1. Introduction

Multiple sclerosis (MS) is a chronic inflammatory disease of the central nervous system that affects more than 2.8 million people worldwide [1]. It is an immune-mediated disease in which the myelin sheath that protects the nerve cells in the brain and spinal cord is impaired, making MS the leading cause of disability in young and middle-aged adults [2,3].

Throughout the second half of the 20th century, incidence and prevalence of MS have increased. Many studies showed that the epidemiological characteristic of MS has an irregular distribution of the disease across the world [4]. The interplay between geographical, environmental and the socioeconomic structure, but also the population genetics, had shown to have a major role in the MS distribution [5]. This disease is more prevalent in Nordic and Temperate regions, particularly in high-income countries, while its prevalence is lower in tropical zones and low-income countries [6]. The underlying cause of the latitudinal gradient is not completely understood, but the genetic and environmental factors appear to play a significant role [7].

The epidemiological landscape of MS follows a well-documented latitudinal gradient in Europe, which significantly influences disease prevalence and clinical presentation. Denmark reports one of the highest prevalence rates globally, estimated at approximately 300 per 100,000 inhabitants, whereas Romania presents a lower, albeit rising, crude prevalence of approximately 53.6 per 100,000. These disparities are driven by a complex interplay of genetic susceptibility and environmental factors, including geographic latitude and ultraviolet radiation exposure (sunlight).

Beyond latitude, several modifiable risk factors are critical determinants of disease onset and progression. Low serum Vitamin D levels, history of Epstein–Barr virus (EBV) infections, and lifestyle behaviors such as tobacco intake, obesity and alcohol abuse have been identified as key contributors to neuroinflammation [8]. Emerging evidence suggests that nutritional patterns play a synergistic role; adherence to a Mediterranean diet is associated with lower MS severity and a reduced risk of disease progression by modulating oxidative stress and gut microbiota [9,10]. In contrast, a ‘Western diet’—rich in ultra-processed foods and saturated fats—is linked to increased systemic inflammation and a higher likelihood of worse clinical outcomes [11].

Despite recent advances in disease-modifying therapies (DMTs), there remains a marked scarcity of knowledge regarding how these environmental and structural variables influence clinical phenotypes in diverse geographical settings. Most of the existing literature focuses on single-center or national cohorts, providing limited comparative data between established Nordic health systems and emerging Eastern European regional centers.

Studying the incidence and prevalence of a disease provides valuable insights into its risk factors, helps optimize therapeutic strategies, aids in planning economic costs and evaluates the social impact of the disease [12].

In recent years, the management of MS has undergone a significant paradigm shift, moving away from the traditional ‘escalation’ or ‘step-care’ approach toward the early initiation of high-efficacy disease-modifying therapies (HE-DMTs), such as anti-CD20 monoclonal antibodies and sphingosine-1-phosphate (S1P) receptor modulators [13]. Current evidence suggests that utilizing HE-DMTs early in the disease course is superior in reducing long-term disability accrual and minimizing ‘progression independent of relapse activity’ (PIRA), also known as ‘silent progression’ [14]. However, the implementation of these up-to-date treatment protocols is not uniform globally. According to the WHO Atlas of MS (2020/2022), significant disparities exist in therapeutic access and diagnostic infrastructure between high-income Nordic regions and South-Eastern European countries [15,16]. Understanding how these regional variations in healthcare delivery and treatment timing influence clinical phenotypes is essential for optimizing care and addressing the ‘therapeutic gap’ in different geographical populations. 

The Danish Multiple Sclerosis Registry (DMSR) was established in 1956 throughout the survey conducted by Kay Hyllested, with data collection dating back to 1948. It began as a nationwide, population-based MS follow-up and continues to record new cases, serving as a key resource for epidemiological analyses [17,18]. In 1996, a national database was established to include data of disease-modifying therapies (DMTs), which was later affiliated with the DMSR [19].

In Romania, the Neurology 1 Clinic at the Emergency County Hospital of Târgu Mureș was the first center to provide DMTs for MS, beginning in 2000. From that point onward, patients have been diagnosed, treated and monitored as part of the National MS program [20].

The novelty of this research lies in its large-scale, cross-sectional evaluation patients, providing a unique “natural experiment” between two disparate European healthcare ecosystems. While MS research is frequently dominated by data from single, high-income registries, this study offers a rare head-to-head comparison of clinical phenotypes and management strategies across the North–South latitudinal and West–East socioeconomic axes of Europe.

Unlike the existing literature, which often assumes uniform outcomes under standardized diagnostic criteria, our work explores the “implementation gap” between established Nordic treatment paradigms and the evolving clinical landscapes of South-Eastern Europe. Furthermore, this study is among the first to examine the intersection of regional clinical presentation (specifically the anatomical localization of onset symptoms) and long-term disability status in these distinct populations. By synthesizing these diverse variables, the study provides novel insights into how regional healthcare infrastructure and environmental contexts influence the real-world disease course of MS.

We hypothesize that despite the universal adoption of the 2017 McDonald diagnostic criteria, patients in these two regional hubs will demonstrate divergent clinical trajectories and disability accrual patterns. We anticipate that these differences are not merely biological but are significantly influenced by regional variations in therapeutic infrastructure and distinct environmental contexts, including dietary habits and sunlight exposure.

This study aims to address a critical scarcity in current knowledge by quantifying the “therapeutic gap” between established Nordic management paradigms and emerging Eastern European clinical practices. Furthermore, we investigate the predictive value of initial clinical symptoms, with a focus on pyramidal involvement, as early determinants of long-term disability status in these diverse settings. By aligning clinical data from two disparate healthcare environments, this study seeks to provide a broader understanding of how standardized care protocols translate into real-world outcomes.

## 2. Materials and Methods

This retrospective, observational cohort study compared clinical and demographic profiles of patients with MS patients from two distinct European centers: the Danish Multiple Sclerosis Center (DMSC) at Rigshospitalet, Copenhagen, Denmark, and the Regional MS Center at Neurology 1 Clinic, Târgu Mureș County Hospital, Romania. The study period spanned from 1 January 2010 to 31 December 2024.

A total of 3501 patients were included in this comparative analysis, comprising patients from the DMSC (*n* = 2760) and patients from the Regional MS Center in Târgu Mureș (*n* = 741). This cohort represents the total consecutive treated population at both tertiary referral centers that met the inclusion criteria during the study period.

Data for Danish patients were obtained from the Danish Multiple Sclerosis Registry (DMSR), which is a high-validity, clinician-driven registry where data collection is mandatory during routine medical follow-up on all persons with demyelinating diseases.

Romanian patients’ data were extracted from the Regional MS Center in Târgu Mureș, Neurology 1 Clinic, which has been monitoring and treating MS patients through the National MS program since 2000. This electronically managed database maintains individual patient records that are regularly updated by attending neurologists following from each patient’s visit. We extracted and compared demographic and clinical information data from both data sources.

To establish the study population, all living patients on active disease modifying therapy (DMT) between 2010 and 2024 at the DMSC from Copenhagen University Hospital Rigshospitalet and Regional MS Center in Târgu Mureș were included. Inclusion was exhaustive, encompassing all treated individuals who met the 2017 McDonald diagnostic criteria and had available clinical follow-up data. This approach was chosen to minimize selection bias and ensure that the study population accurately reflects the real-world clinical practice in both Denmark and Romania. To ensure a homogenous study population, a rigorous differential diagnostic screening was applied to all medical records. Patients primarily diagnosed with other central nervous system inflammatory demyelinating diseases, specifically Neuromyelitis Optica Spectrum Disorder (NMOSD) and Myelin Oligodendrocyte Glycoprotein Antibody-associated Disease (MOGAD), were systematically identified and excluded from the final analysis. This process ensured that the resulting cohort consisted strictly of patients meeting the 2017 McDonald criteria for Multiple Sclerosis, thereby minimizing clinical and phenotypic confounding from non-MS pathologies.

Patients were stratified into predefined age groups by decades. We evaluated the mean and median age at symptom onset and MS diagnosis. Furthermore, the time between the first symptom and the diagnosis was determined. The disease was classified by phenotype as relapsing-remitting multiple sclerosis (RRMS), secondary-progressive multiple sclerosis (SPMS), primary-progressive multiple sclerosis (PPMS). For each disease phenotype, the percentage of patients in each center was calculated. All included patients were clinically assessed using the EDSS, allowing us to calculate the mean disability score and categorize patients based on their EDSS score (0–2.5, 3–5.5, >6). Patients enrolled from 2010 onward were initially assessed according to the 2010 McDonald criteria, with diagnostic classification subsequently updated using the 2017 McDonald criteria [21,22].

Onset symptoms were categorized (sensory, visual, pyramidal, brainstem, cerebellar, bladder/bowel, multifocal, other, unknown) to evaluate differences in disease presentation between the two clinics.

DMT were subdivided into three groups: high-efficacy treatment (Alemtuzumab, HSCT, Natalizumab, Ocrelizumab, Ofatumumab, Rituximab, Treosulfan, Daclizumab, Mitoxantrone), medium-efficacy treatment (Azathioprin, Cladribine, Dimethyl fumarate, Diroximel fumarate, Fingolimod, Methotrexate, Mycophenolatmofetil, Ozanimod, Siponimod, Laquinimod) and low-efficacy treatment (Interferon Beta-1A, Beta-1B and pegylated, Teriflunomide, Glatiramer acetate). We calculated the percentage of patients receiving each treatment, assessed whether they had previously been treated with another DMT, and analyzed the reasons for treatment discontinuation.

## 3. Statistical Analysis

Demographic and clinical characteristics of the study cohorts were summarized according to variable type. Categorical variables are expressed as frequencies and corresponding percentages. Continuous variables are presented as mean ± standard deviation (SD) for normally distributed data and as median with interquartile range (IQR) for non-normally distributed data.

The Wilcoxon rank-sum test was used to compare median values between two independent groups (e.g., age, disease duration, age at MS onset, age at MS diagnosis, time between MS onset and diagnosis and EDSS). The Chi-square test with simulated *p*-values was applied to assess associations between categorical variables (e.g., age categories, sex, MS phenotype, onset symptoms, current DMTs and reasons for discontinuation). Due to the non-normal distribution of clinical variables (notably EDSS scores) and the inherent imbalance between the two geographical cohorts, non-parametric statistical methods were prioritized. The Wilcoxon rank-sum test was employed for continuous variables, as it is robust against skewed distributions and unequal group sizes. Categorical comparisons were performed using the Chi-square test with simulated *p*-values to ensure statistical accuracy despite the population size discrepancy. These methods allow for a rigorous comparison without the need for data transformation, which can obscure the clinical interpretability of MS disability scales.

All statistical analyses were performed using R software (version 4.1.0; R Foundation for Statistical Computing, Vienna, Austria) [23].

The study was conducted in accordance with the Declaration of Helsinki and the required informed consent was obtained from participants prior to inclusion in the study (ethical approval nr. Ad. 7749/5 April 2023, F-PS-0113-07).

## 4. Results

A comparative analysis of baseline demographics revealed high consistency between the DMSC (N = 2760) and the Târgu Mureș cohort (*n* = 741). Gender distribution was balanced across both regions, with a characteristic female predominance (DMSC: 69.2%; Târgu Mureș: 65.7%). The age profiles at clinical onset and diagnosis were nearly identical, with a mean onset age of 32 years and a mean diagnosis age of 34 years in both populations.

Statistical evaluation of the diagnostic lag showed a mean interval between symptom onset and clinical confirmation of 2.78 years for DMSC and 2.15 years for Târgu Mureș. Table 1 indicates *p* > 0.05, these data suggest that both cohorts follow a similar chronological trajectory from first symptom to formal diagnosis, establishing a comparable baseline for the subsequent evaluation of phenotype and disability progression.

**Figure 1:**
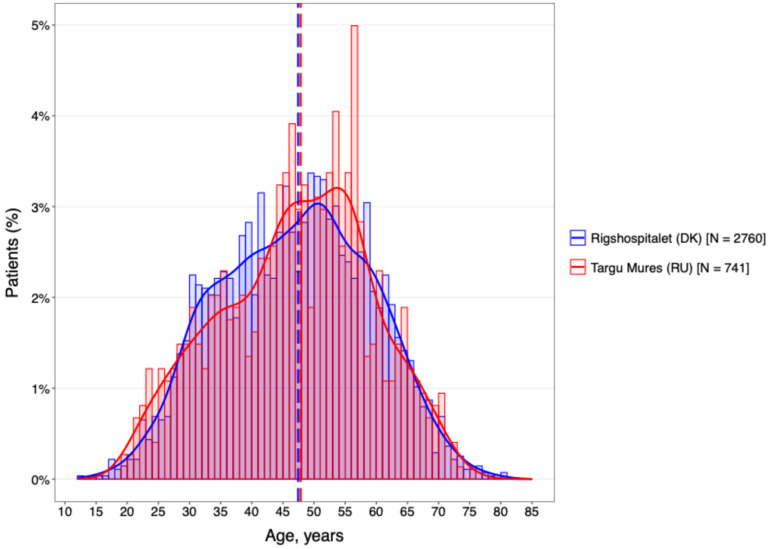
DMT by age categories.

Analysis of the age distribution (Figure 1) revealed a notable demographic shift between the two cohorts. While the DMSC population was predominantly composed of younger patients in the 35–45 year age bracket, the Târgu Mureș cohort was characterized by a significantly older on-treatment population, peaking between 45 and 55 years.

This rightward shift in the Romanian age distribution suggests a higher proportion of patients with longer disease durations remaining under active clinical follow-up. Given the established link between chronological age and the transition from inflammatory to neurodegenerative disease phases, this age discrepancy likely serves as a primary driver for the increased disability levels and higher prevalence of progressive phenotypes observed in the Romanian group.

**Figure 2:**
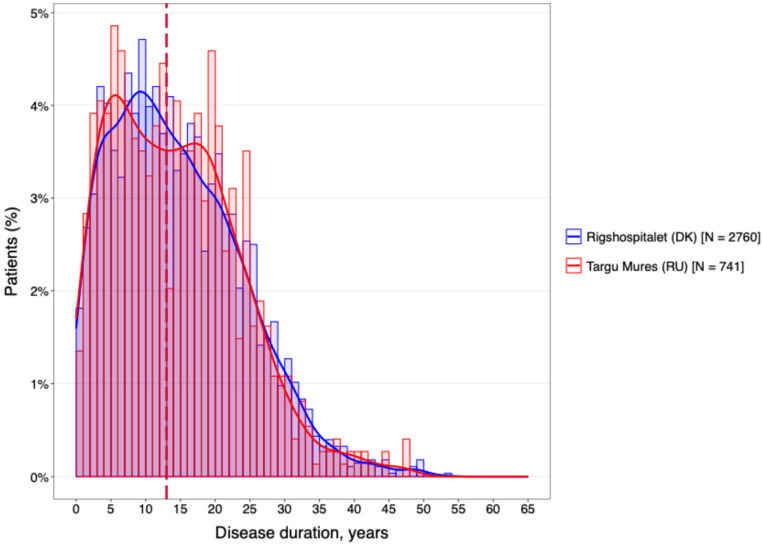
The mean of disease duration of the MS population for both centers.

**Figure 3:**
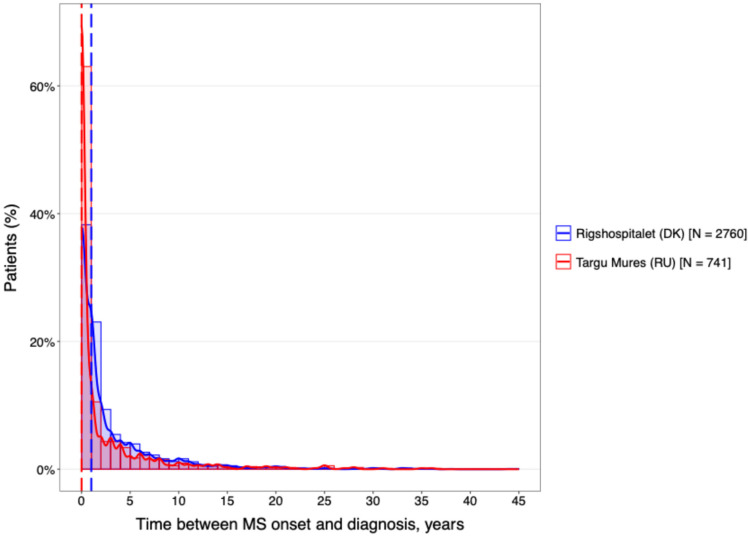
The difference between the diagnostic delay.

The temporal characteristics of the disease were remarkably consistent across both geographical regions (Figure 2). The cohorts were well-matched for disease chronicity, with a mean duration of 14.4 years at the DMSC and 14.2 years in Târgu Mureș. Furthermore, analysis of the diagnostic delay (Figure 3) revealed no statistically significant difference between the two centers (*p* > 0.05).

The clinical characteristics and disability profiles of the two cohorts are summarized in Table 2. While the phenotypic distribution was statistically comparable with a high predominance of RRMS in both DMSC (87.4%) and Târgu Mureș (90.0%), a significant divergence emerged in disability status. At the time of analysis, the mean EDSS score was notably higher in the Târgu Mureș cohort (3.47) compared to the DMSC (2.59).

**Figure 4:**
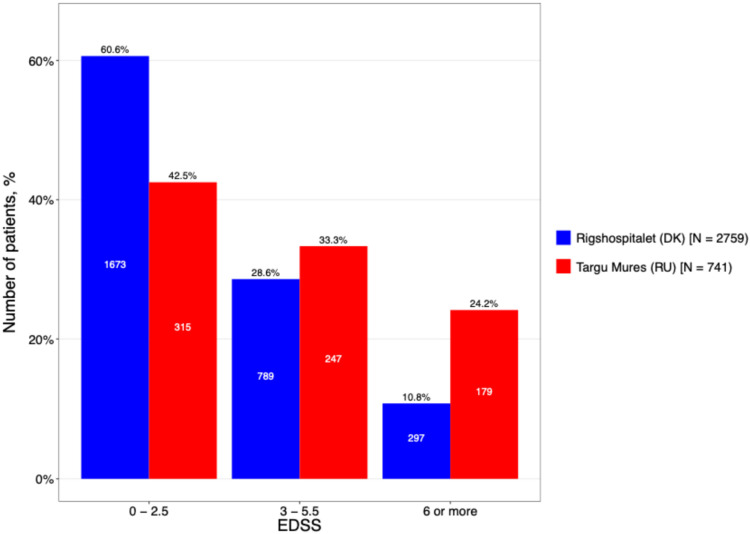
The current EDSS level stratification by center.

To further characterize this disability gap, patients were stratified into mild (0–2.5), moderate (3.0–5.5) and severe (≥6.0) EDSS subgroups (Figure 4). The DMSC cohort was predominantly represented in the mild disability subgroup (60.6%), whereas the Târgu Mureș cohort showed a higher concentration of patients in the moderate and severe categories. This shift toward higher EDSS scores in the Romanian population suggests a more rapid accrual of disability or a more aggressive disease trajectory, despite the similar total disease duration reported earlier.

**Figure 5:**
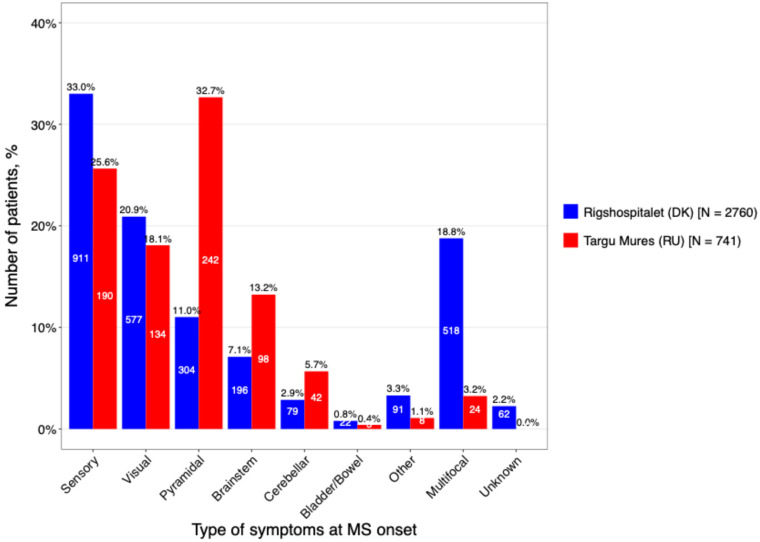
The main types of onset symptoms stratified by caring center.

Distinct patterns were also observed in symptom presentation at onset (Figure 5). In Târgu Mureș, pyramidal symptoms were the primary presenting feature (32.7%), indicating early motor pathway involvement. In contrast, DMSC patients more frequently presented with sensory symptoms (33%) or a multifocal onset (18.8%). This higher prevalence of early pyramidal signs in the Romanian cohort is analytically significant, as motor symptoms at onset are established clinical predictors of a poorer long-term prognosis and faster transition to secondary progression.

**Figure 6:**
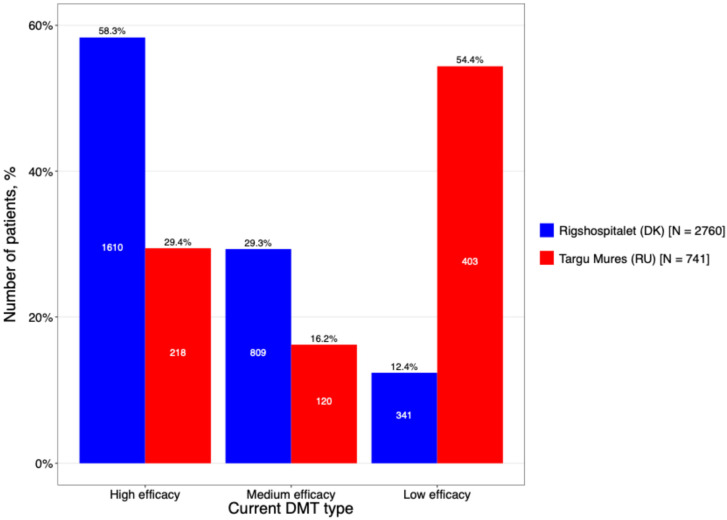
The DMT treatment categorized based on its efficacy (high efficacy include Alemtuzumab, HSCT, Natalizumab, Ocrelizumab, Ofatumumab, Rituximab, Treosulfan, Daclizumab, Mitoxantrone; Medium efficacy include Azathioprin, Cladribine, Dimethyl fumarate, Diroximel fumarate, Fingolimod, Methotrexate, Mycophenolat mofetil, Ozanimod, Siponimod, Laquinimod. Low efficacy include Interferon (beta-1a, beta-1b and pegylated), Teriflunomide, Glatiramer acetate).

A comparative analysis of DMT utilization revealed divergent treatment strategies between the two centers (Figure 6). The Romanian cohort was characterized by a traditional escalation approach, with a heavy reliance on first-generation injectables; specifically, interferon-β preparations were used as first-line therapy at nearly double the rate of the Danish cohort (74.1% vs. 42.5%). In contrast, the DMSC cohort demonstrated a more proactive transition to modern treatment paradigms, with significantly higher utilization of both oral therapies (20.0% vs. 7.0%) and high-efficacy DMTs (7.4% vs. 2.2%).

These differences extended to the sustainability of the therapeutic interventions. Treatment duration was significantly longer in the Danish cohort (67.8 ± 34.2 vs. 58.3 ± 29.7 months), suggesting better long-term persistence. Conversely, the Romanian cohort exhibited a significantly higher discontinuation rate (44.3% vs. 35.1%, *p* = 0.004). The primary driver for cessation in both groups was the occurrence of adverse events, though this was more pronounced in the Romanian sample (50.0% vs. 42.9%). As summarized in Table 3, the combination of a more restrictive therapeutic range and higher intolerance to legacy injectables in the Târgu Mureș cohort suggests a more challenging management landscape, which may contribute to the disability accrual observed in this population.

**Figure 7:**
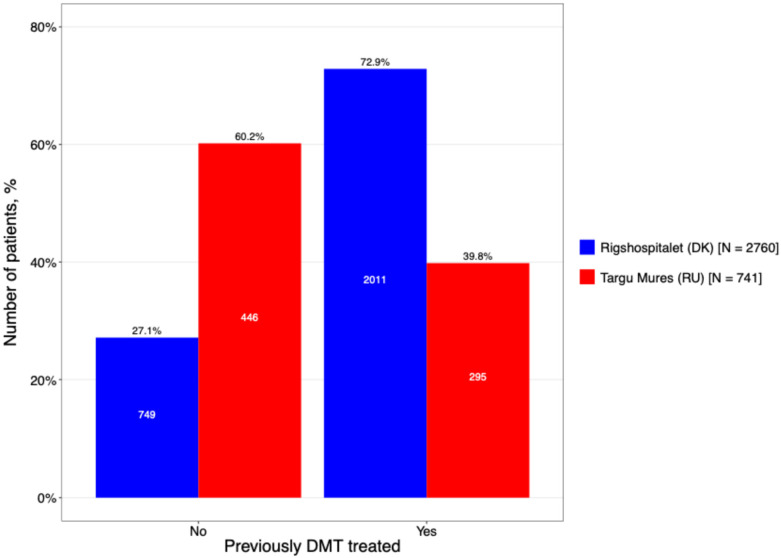
Proportion of patients on treatment with prior DMT exposure.

A significant disparity was identified regarding prior DMT exposure, reflecting divergent clinical histories at the time of inclusion (Figure 7). The DMSC cohort was characterized by a high degree of therapeutic experience, with 72.9% of patients having received previous treatment. In contrast, the Târgu Mureș cohort was predominantly treatment-naïve (60.2%). This suggests that the Romanian center captured a larger proportion of patients earlier in their therapeutic journey or during the initiation of their first-line registry-tracked DMT.

Among the subgroup of patients with prior therapy, the escalation model was evident. In Târgu Mureș, prior exposure was heavily dominated by Interferon-beta (43.1%), appearing at more than double the rate seen in the DMSC (19.0%). Interestingly, both centers showed similar utilization of high-efficacy options prior to switching, such as Natalizumab (15.9% in Târgu Mureș vs. 18.1% in DMSC). This indicates that while first-line choices differ significantly by region, both centers demonstrate a shared clinical threshold for transitioning to high-potency monoclonal antibodies when initial therapies fail.

## 5. Discussion

This comparative study included a total of 3501 patients, from two distinct European centers, revealing significant demographic and clinical differences between Danish and Romanian MS populations. Our findings demonstrate that patients from the DMSC are older at disease onset, receive more diverse DMT, experience longer treatment duration, compared to their Romanian counterparts. These differences likely reflect the complex interplay of genetic, environmental, healthcare system and socioeconomic factors that influence MS epidemiology and management across different European regions. According to the Atlas of MS, the prevalence of MS in Denmark is >200 individuals per 100,000 people, while in Romania it is 26–50 individuals per 100,000 people [24].

Changes in the incidence and prevalence of MS are significantly influenced by advancements in diagnostic capabilities and criteria. The late 20th and early 21st centuries saw the widespread integration of MRI technology, the development of more sensitive diagnostic criteria, such as the McDonald criteria and the introduction of DMTs. These innovations have enabled the earlier and more frequent detection of MS, which has, in turn, led to an increase in reported cases. Essentially, the rise in incidence and prevalence is at least partially an artifact of improved diagnostic yield, identifying cases that would have previously been undiagnosed or diagnosed at a later stage [25].

Both populations demonstrated the expected female predominance (Denmark 2.0:1, Romania 2.4:1), consistent with global MS epidemiology [26].

The compared cohorts showed no statistically significant differences in disease duration, age at disease onset (32.0 years in the Danish cohort vs. 32.4 years in the Romanian cohort), age at MS diagnosis (34.8 years vs. 34.6 years, respectively).

While the total disease duration was comparable between the two groups, our analysis revealed a significant disparity in the latency to diagnosis. As shown in Table 1, the Romanian cohort experienced a significantly longer interval between symptom onset and clinical confirmation. This suggests a narrower ‘window of therapeutic opportunity’ for these patients. Even within a similar total disease timeframe, a prolonged duration of untreated inflammatory activity prior to the initiation of DMTs likely facilitates early axonal damage and disability accrual. This ‘diagnostic lag’ may explain the higher prevalence of pyramidal signs and elevated EDSS scores observed in the Romanian cohort.

Accurate and timely diagnosis of MS is essential to ensure appropriate patient management. The characterization of demyelinating disease relies on a detailed medical history, thorough neurological examination, and the exclusion of alternative diagnoses. The revised 2024 McDonald criteria offer guidance that enables neurologists to achieve a more rapid and precise diagnosis, even in the presence of non-specific symptoms [27].

The similar distribution of MS phenotypes between centers (87.4% vs. 90.0% RRMS) suggests that fundamental disease biology remains consistent across geographic regions, despite environmental and genetic differences.

The proportion of patients with RRMS was comparable between cohorts; however, SPMS was more frequent in the Romanian cohort (19.2%) compared with the Danish cohort (8.1%). Differences were also observed in initial presenting symptoms, with pyramidal signs being most common among Romanian patients (32.7%), whereas sensory signs predominated in the Danish cohort (33%). Differences between the Romanian and Danish groups appear to be driven by both disease characteristics and healthcare system dynamics. The higher disability burden in the Romanian cohort (more SPMS, higher EDSS) suggests potential challenges such as delayed diagnosis over time, possibly related to late presentation to medical care overall. Meanwhile, the distinct initial symptoms, motor signs in Romania versus sensory signs in Denmark, may reflect regional variation in disease presentation or differences in clinical recognition. The significantly higher prevalence of pyramidal signs at onset in the Romanian cohort is a critical finding. Pyramidal involvement (e.g., spasticity, motor weakness) is a known marker for spinal cord involvement and a predictor of poorer long-term disability outcomes. This phenotypic difference further explains why the Romanian cohort presented with higher baseline EDSS scores.

Overall, these results demonstrate that geographic and healthcare system factors must be considered when interpreting data on disease progression between different populations.

This study included exclusively treated patients from both centers. While in Romania the introduction of immunomodulatory therapy MS began in 2000 with the administration of interferon beta-1b at Neurology Clinic 1 of the Emergency County Hospital in Târgu Mureș, a pioneering initiative had already taken place in Denmark [28,29].

In recent years, the emergence of multiple breakthrough therapies for MS has facilitated the use of high- to medium-efficacy agents early in the disease course [30].

The most striking difference in treatment approaches between the two sites: 58.3% of treatments at DMSC were high-efficacy DMTs, contrasting with Târgu Mureș County Hospital, where low-efficacy DMTs were the primary choice (54.4%).

For many individuals with MS on long-term first-line DMTs, the decision to continue the same therapy often reflects both personal preference and established management practices. These agents, while classified as first-line, effectively control disease activity, and patients often develop a sense of stability with their treatment. Concerns about potential risks and side effects of alternative therapies further reinforce the choice to maintain the current DMT. This is reflected in our cohort, where low-efficacy DMTs predominated at Târgu Mureș County Hospital, in contrast to DMSC, where high-efficacy therapies were more commonly used, highlighting differences in treatment strategies, patient preferences, and overall management approaches between the centers [31]. The main avenue for receiving DMTs is through the Romanian National Programme for Treatment of Neurological Diseases. This program ensures that DMTs are fully reimbursed for eligible patients, although accessibility can still be challenging for those not living near one of the 15 dedicated MS centers. Furthermore, a major challenge noted in policy reports is the significant delay (sometimes more than 4 years) between the European Medicines Agency (EMA) approval and the final national approval and reimbursement inclusion for new therapies. This means that while a drug might be approved in Europe in a certain year, Romanian patients may wait several years for it to become widely accessible through the national program [32].

The lower prevalence of DMT utilization in the Romanian cohort (Târgu Mureș) relative to the Danish cohort likely reflects historical disparities in national reimbursement frameworks and the delayed timeline of therapeutic approvals in South-Eastern Europe. This ‘therapeutic gap’ represents a critical period of untreated inflammatory activity which, as addressed in our study limitations, potentially facilitates the increased disability burden and higher EDSS scores observed in this population.

Our study highlights the critical role of reliable real-world data sources, such as population-based registries, in generating precise and valid epidemiological measures from well-defined study populations [33]. Such data are essential for characterizing the evolving prognosis of MS and the most efficient treatment that could delay the disease progression [34].

While this study provides a large-scale view of two regional MS populations, it is primarily a comparative analysis of two distinct clinical case series rather than a true population-wide cross-sectional study; as such, it lacks a healthy control group for comparison. Consequently, the findings from these specific urban centers, Copenhagen and Târgu Mureș, may not be fully generalizable to the total national MS populations or account for rural–urban health disparities within each country. Furthermore, as a retrospective registry-based study, we lacked individual-level data on critical environmental risk factors such as history of infections, obesity, tobacco intake and adherence to a Mediterranean diet, all of which are established modulators of disease progression [35,36].

Moreover, the lack of stratification by diagnosis era, precluded by our sample size, means we cannot entirely exclude the influence of historical treatment landscapes. The observed differences in disease severity, specifically the higher EDSS scores in the Romanian cohort, should therefore be interpreted with caution, as they may be confounded by varying historical access to high-efficacy DMTs and differing regional socioeconomic conditions [37,38]. Additionally, the absence of systematic, longitudinal MRI data prevented a detailed radiological comparison of lesion burden. Finally, our cohort consists exclusively of treated patients currently under active follow-up; this introduces a potential selection bias, as patients who discontinued clinical visits could not be re-evaluated to update their neurological status or disability progression [39,40].

## 6. Conclusions

This study reveals both common and distinct characteristics among MS patients treated at the two centers, emphasizing the influence of regional factors on disease management and therapeutic decision-making. This comparative analysis of 3501 patients reveals that despite nearly identical disease durations, MS patients at the Târgu Mureș regional center exhibit a significantly higher disability burden and a more severe initial clinical phenotype compared to those at the DMSC. This study highlights both shared and distinct characteristics among patients at these two centers, emphasizing that the clinical trajectory of MS is profoundly influenced by the regional therapeutic landscape and healthcare infrastructure.

The higher prevalence of low-efficacy DMT use in the Romanian cohort, contrasted with the earlier and more frequent adoption of high-efficacy paradigms in Denmark, appears to be a primary driver of the observed “disability gap”. Our findings underscore that variations in treatment availability and clinical practice patterns contribute significantly to divergent patient outcomes. Recognizing these regional disparities is essential for optimizing care and ensuring equitable access to high-efficacy therapies across Europe.

Ultimately, developing tailored management strategies that reflect local healthcare contexts and therapeutic access is necessary to minimize long-term disability. These results suggest that standardized diagnostic criteria alone are insufficient to harmonize patient outcomes without a corresponding alignment in therapeutic intervention strategies across different regional centers.

## Figures and Tables

**Table 1:** Demographic and clinical characteristics of on-treatment MS patients by caring center.

		Center		*p*-Value
		DMSC (DK) (N = 2760)	Târgu Mureș (RO) (N = 741)	
**Age, n (%)**				0.98 χ
	**0–19**	17 (0.6%)	2 (0.3%)	
	**20–29**	209 (7.6%)	73 (9.9%)	
	**30–39**	621 (22.5%)	133 (17.9%)	
	**40–49**	739 (26.8%)	205 (27.7%)	
	**50–59**	750 (27.2%)	223 (30.1%)	
	**60–69**	367 (13.3%)	93 (12.6%)	
	**70–79**	55 (2.0%)	12 (1.6%)	
	**80–89**	2 (0.1%)	0 (0%)	
**Sex, n (%)**				0.551 χ
	**Female**	1909 (69.2%)	487 (65.7%)	
	**Male**	851 (30.8%)	254 (34.3%)	
**Mean age at MS onset (SD)** **Median [Q1, Q3] age at MS onset**				0.72 ^w^
	-	32.0 (9.97) 31.0 [25.0, 38.0]	32.4 (9.94) 31.0 [25.0, 40.0]	
**Mean Age at MS diagnosis (SD)** **Median [Q1, Q3] age at MS diagnosis**				0.854 ^w^
	-	34.8 (10.5) 33.0 [27.0, 42.0]	34.6 (10.4) 34.0 [26.0, 42.0]	
**Mean time between MS onset and diagnosis (SD)** **Median [Q1, Q3] time between MS onset and diagnosis**				0.00112 ^w^
	-	2.78 (4.82) 1.00 [0, 3.00]	2.15 (4.89) 0 [0, 2.00]	
**Mean of disease duration (SD)** **Median [Q1, Q3] of disease duration**				0.819 ^w^
	-	14.4 (9.38) 13.0 [7.00, 21.0]	14.2 (9.23) 13.0 [6.00, 20.0]	

DK: Denmark; RO: Romania; MS: multiple sclerosis; SD: standard deviation; Q1, Q3: interquartile range; ^w^ = Wilcoxon rank sum test.; χ = Chi-square test with simulated *p*-values.

**Table 2:** Clinical characteristics of on-treatment MS patients stratified by caring center.

		Center		*p*-Value
		DMSC (DK) (N = 2760)	Târgu Mureș (RO) (N = 741)	
**MS phenotype, n (%)**				0.005 χ
	**RRMS**	2485 (90.0%)	574 (77.5%)	
	**SPMS**	224 (8.1%)	142 (19.2%)	
	**PPMS**	51 (1.8%)	25 (3.4%)	
**EDSS, n (%)**				<0.001 χ
	**0–2.5**	1673 (60.6%)	315 (42.5%)	
	**3–5.5**	789 (28.6%)	247 (33.3%)	
	**>6**	297 (10.8%)	179 (24.2%)	
**Onset symptoms, n (%)**				<0.001 χ
	**Sensory**	911 (33.0%)	190 (25.6%)	
	**Visual**	577 (20.9%)	134 (18.1%)	
	**Pyramidal**	304 (11.0%)	242 (32.7%)	
	**Brainstem**	196 (7.1%)	98 (13.2%)	
	**Cerebellar**	79 (2.9%)	42 (5.7%)	
	**Bladder/bowel**	22 (0.8%)	3 (0.4%)	
	**Multifocal**	518 (18.8%)	24 (3.2%)	
	**Other**	91 (3.3%)	8 (1.1%)	
	**Unknown**	62 (2.2%)	0 (0%)	

EDSS: Expanded Disability Status Scale; RRMS: relapsing-remitting multiple sclerosis; SPMS: secondary progressive multiple sclerosis; PPMS: primary progressive multiple sclerosis; DK: Denmark; RO: Romania; χ = Chi-square test with simulated *p*-values.

**Table 3:** Treatment of MS patients stratified by caring center.

		Center		*p*-Value
		DMSC (DK) (N = 2760)	Târgu Mureș (RO) (N = 741)	
Current DMT *, n (%)				<0.001 χ
	**High efficacy**	1610 (58.3%)	218 (29.4%)	
	**Medium efficacy**	809 (29.3%)	120 (16.2%)	
	**Low efficacy**	341 (12.4%)	403 (54.4%)	
**Previously treated, n (%)**				<0.001 χ
	**No**	749 (27.1%)	446 (60.2%)	
	**Yes**	2011 (72.9%)	295 (39.8%)	
**Last DMT, n (%)**				0.86 χ
	**Aleztuzumab**	26 (1.3%)	0 (0%)	
	**Azathioprin**	4 (0.2%)	0 (0%)	
	**Cladribine**	43 (2.1%)	1 (0.3%)	
	**Daclizumab**	12 (0.6%)	2 (0.7%)	
	**Dimethyl fumarate**	282 (14.0%)	16 (5.4%)	
	**Diroximel fumarate**	2 (0.1%)	0 (0%)	
	**Fingolimod**	250 (12.4%)	11 (3.7%)	
	**Glatiramer acetate**	179 (8.9%)	40 (13.6%)	
	**HSCT**	11 (0.5%)	0 (0%)	
	**Interferon**	383 (19.0%)	127 (43.1%)	
	**Laquinimod**	0 (0%)	2 (0.7%)	
	**Methotrexate**	13 (0.6%)	0 (0%)	
	**Mitoxantrone**	10 (0.5%)	0 (0%)	
	**Natalizumab**	364 (18.1%)	47 (15.9%)	
	**Ocrelizumab**	129 (6.4%)	5 (1.7%)	
	**Ofatumumab**	43 (2.1%)	1 (0.3%)	
	**Ozanimode**	2 (0.1%)	0 (0%)	
	**Rituximab**	36 (1.8%)	0 (0%)	
	**Siponimod**	1 (0.0%)	4 (1.4%)	
	**Teriflunomide**	212 (10.5%)	39 (13.2%)	
	**Treosulfan**	9 (0.4%)	0 (0%)	
**Discontinuation Reason,** **n (%)**				0.0335 χ
	**Adverse events**	390 (19.5%)	66 (22.4%)	
	**Disease activity**	731 (36.6%)	116 (39.3%)	
	**JC Virus**	196 (9.8%)	31 (10.5%)	
	Other reason **	359 (18.0%)	3 (1.0%)	
	**Patients’ decision**	58 (2.9%)	9 (3.1%)	
	**Practical issues**	46 (2.3%)	2 (0.7%)	
	**Pregnancy**	152 (7.6%)	10 (3.4%)	
	**Progression**	64 (3.2%)	58 (19.7%)	

* High efficacy: Alemtuzumab, HSCT, Natalizumab, Ocrelizumab, Ofatumumab, Rituximab, Treosulfan, Daclizumab, Mitoxantrone/Medium efficacy: Azathioprin, Cladribine, Dimethyl fumarate, Diroximel fumarate, Fingolimod, Methotrexate, Mycophenolatmofetil, Ozanimod, Siponimod, Laquinimod/Low efficacy: Interferon (beta-1A, beta-1B and pegylated), Teriflunomide, Glatiramer acetate. ** Other reason category include anti-drug antibodies formation; stable condition; contra indication; lack of patient compliance; pregnancy/breastfeeding; terminated course. DK: Denmark; RO: Romania; DMT: disease modifying treatment; HSCT: hematopoietic stem cell transplantation; JC Virus: Johm Cunningham virus; χ = Chi-square test with simulated *p*-values.

## Data Availability

The original contributions presented in this study are included in the article. Further inquiries can be directed to the corresponding author.
